# Rapid gene fusion testing using the NanoString nCounter platform to improve pediatric leukemia diagnoses in Sub-Saharan Africa

**DOI:** 10.3389/fonc.2024.1426638

**Published:** 2024-06-13

**Authors:** Julie M. Gastier-Foster, Fredrick Lutwama, Olive Mbabazi, Steven Mlenga, Kennedy Ulaya, Ruth Namazzi, E. Faith Hollingsworth, Dolores Lopez-Terrada, Kevin E. Fisher, Angshumoy Roy, Carl E. Allen, David G. Poplack, Rizine Mzikamanda, Nmazuo Ozuah, Peter Wasswa

**Affiliations:** ^1^ Global HOPE, Texas Children’s Hospital, Houston, TX, United States; ^2^ Department of Pathology, Texas Children’s Hospital, Houston, TX, United States; ^3^ Department of Pediatrics, Baylor College of Medicine, Houston, TX, United States; ^4^ Department of Pathology & Immunology, Baylor College of Medicine, Houston, TX, United States; ^5^ Biomedical Research Centre, Makerere University, Kampala, Uganda; ^6^ Baylor College of Medicine Children’s Foundation - Malawi, Lilongwe, Malawi; ^7^ College of Health Sciences, Makerere University, Kampala, Uganda

**Keywords:** pediatric leukemia, childhood leukemia, Africa, LMIC, molecular diagnosis, gene fusion, nanostring nCounter, genomics

## Abstract

Risk stratification and molecular targeting have been key to increasing cure rates for pediatric cancers in high-income countries. In contrast, precise diagnosis in low-resource settings is hindered by insufficient pathology infrastructure. The Global HOPE program aims to improve outcomes for pediatric cancer in Sub-Saharan Africa (SSA) by building local clinical care and diagnostic capacity. This study aimed to assess the feasibility of implementing molecular assays to improve leukemia diagnoses in SSA. Custom NanoString nCounter gene fusion assays, previously validated in the US, were used to test samples from suspected leukemia patients. The NanoString platform was chosen due to relatively low cost, minimal technical and bioinformatics expertise required, ability to test sub-optimal RNA, and rapid turnaround time. Fusion results were analyzed blindly, then compared to morphology and flow cytometry results. Of 117 leukemia samples, 74 were fusion-positive, 30 were negative, 7 were not interpretable, and 6 failed RNA quality. Nine additional samples were negative for leukemia by flow cytometry and negative for gene fusions. All 74 gene fusions aligned with the immunophenotype determined by flow cytometry. Fourteen samples had additional information available to further confirm the accuracy of the gene fusion results. The testing provided a more precise diagnosis in >60% of cases, and 9 cases were identified that could be treated with an available tyrosine kinase inhibitor, if detected at diagnosis. As risk-stratified and targeted therapies become more available in SSA, implementing this testing in real-time will enable the treatment of pediatric cancer to move toward incorporating risk stratification for optimized therapy.

## 1 Introduction

In high-resource countries, modern treatment approaches have improved the cure rate for childhood cancers to approximately 85% (1). Achieving these excellent cure rates depends on therapeutic regimens tailored to the specific histological or molecular characteristics of each cancer. Unfortunately, the majority (~80%) of children with cancer live in low- and middle-income countries (LMICs), where there is limited access to pathology services, and cure rates are closer to 20% (1–5). The gold standard of modern cancer diagnosis in Western countries incorporates molecular diagnosis, specifically genomic analysis of the cancer sample. Genomic analysis can be used to determine a precise diagnosis, suggest prognosis to guide risk-adapted treatment, and identify cases that may benefit from targeted therapy. The pediatric cancer treatment protocols used in sub-Saharan Africa (SSA) are typically one treatment regimen per cancer type. This approach means many African children are undertreated and fail to achieve cure, while others are potentially over-treated and unnecessarily exposed to toxic chemotherapy agents.

The Texas Children’s Hospital Global HOPE (Hematology Oncology Pediatric Excellence) program launched in February 2016 as a capacity-building initiative, strengthening local healthcare infrastructure in SSA to effectively provide the multi-disciplinary care that is needed to optimally care for children with cancer, sickle cell disease and other blood disorders (6). The program, based at Texas Children’s Hospital and Baylor College of Medicine, partners with Ministries of Health and local hospitals in SSA. Global HOPE supported the establishment of the first accredited specialist training program for childhood cancer in the East African region (7). More than 30 pediatric hematologists/oncologists have graduated from the program. To improve patient outcomes, it is imperative to develop improved but sustainable diagnostic capacity alongside specialized clinical care for pediatric cancer.

Leukemia is the most common pediatric cancer, accounting for approximately 30% of all cases globally (2), and a similar rate has been observed at the Global HOPE-affiliated sites in Africa. Immunophenotyping is a critical diagnostic method for hematologic cancers in Western settings, and Global HOPE is working to ensure flow cytometry is incorporated at all its affiliated sites in SSA (8). The current approach to leukemia diagnosis in SSA includes morphology review and immunophenotype by flow cytometry (if available). Cytogenetic or molecular genetic analysis is rarely available. Diagnoses based on these limited evaluations often lack the specificity necessary to inform the application of effective, contemporary risk-adapted treatment regimens that incorporate molecular and genomic data. The genomics of pediatric leukemias have been well-studied in high resource settings (9–11). Gene fusions are estimated to occur in >50% of pediatric leukemias (12–15), with an additional 25% due to aneuploidy. The presence of a gene fusion in pediatric leukemia can be prognostic: examples include a favorable prognosis for B-acute lymphoblastic leukemia (B-ALL) with a *ETV6-RUNX1* fusion or unfavorable prognosis of an acute myeloid leukemia (AML) patient with *CBFA2T3-GLIS1* fusion. Specific leukemia fusions can also inform specific therapeutic option such as retinoic acid treatment for AML with a *PML-RARA* fusion, or a tyrosine kinase inhibitor for *BCR-ABL1* or Ph-like B-ALL. Without the addition of a tyrosine kinase inhibitor, the outcome for these B-ALL patients can be as low as 30%, even in high-income countries (16, 17). Given that the highest frequency of genomic changes in pediatric leukemias is due to gene fusions, this class of abnormalities was a logical choice for the introduction of molecular testing in SSA, with the intent to address copy number changes in another round of development.

This project aimed to establish molecular methods to accurately diagnose pediatric cancers in a manner that is sustainable and standardized across multiple sites in the LMICs of SSA. Cost-effective methods for proper diagnosis are a key component to reach this goal and reduce the inequalities of available care. While next-generation sequencing (NGS) analysis has become standard for cancer care in high-income countries, SSA is not yet prepared to utilize NGS due to prohibitive cost, need for highly trained personnel, and intensive bioinformatics requirements. To address the current pathology infrastructure limitations in SSA, it is critical to innovate strategies that consider cost, turnaround time, personnel expertise required, small quantity of tumor available, and means to support interpretation and troubleshooting. Novel high-throughput molecular techniques have been developed with capabilities to detect multiple gene fusions associated with various pediatric cancers. One such platform, the NanoString nCounter analysis system, uses nucleic acid hybridization probes to simultaneously analyze hundreds of gene fusions in a single reaction. This technique requires neither nucleic acid amplification steps, library preparation, nor a bioinformatics pipeline, reducing the need for highly skilled technical or bioinformatics expertise. It is also tolerant of poor nucleic acid quality, making it amenable to a broad range of sample types. This method of fusion analysis requires less than 1 hour of hands-on time for the laboratory technologist, approximately 30 minutes on two successive days. Total time from RNA extraction to results interpretation can be as little as 48 hours, with 12 samples processed per batch. This methodology has been used to develop fusion panels for targeted tumor types (18–22). Given the high frequency of gene fusions in pediatric leukemias and the potential impact to improve outcome via customized therapy, the Global HOPE team tested the feasibility of implementing a custom gene fusion panel at the Global HOPE-affiliated sites in Uganda and Malawi. It was anticipated that the gene fusion results, in combination with the existing morphology and flow cytometry analyses, would be a first step towards refining diagnostic accuracy and eventually improving survival for children with leukemia in SSA.

## 2 Materials and methods

### 2.1 Design and validation of the global HOPE custom gene fusion panels

Literature review, clinical laboratory results and public databases were used to obtain exact breakpoint sequence information for gene fusions associated with pediatric and adolescent cancers. The hematologic malignancy panel was designed to detect 439 breakpoints for 223 non-IGH/TCR fusions reported in ALL, AML, CML, lymphomas, and histiocytosis. The panel was purposely designed to be very broad, incorporating both common and rare gene fusions given the scarcity of knowledge of the genomic landscape of pediatric tumors in SSA. Due to the large size of the panel and limitation of probe sets per multiplexed tube, the Hematologic custom gene fusion panel consisted of two tubes: Heme 1 and Heme 2 with the intent to combine the most relevant probe sets for SSA into a single tube in a future iteration. The fusions in the panel, number of probe sets per fusion, and the number of samples positive for the fusion included in the validation set are shown in **Table 1**. The panels were validated in the Department of Pathology at Texas Children’s Hospital using residual, deidentified pediatric cancer samples with known fusion status determined by previous clinical testing, as well as commercially available RNA controls (Invivoscribe, San Diego, CA, USA and SeraCare Life Sciences, Milford, MA, USA). The validation design included samples to test a variety of fusions, different breakpoints for the same fusion, robustness between technicians, precision across runs, sensitivity of detection, and ease of performance. The NanoString results yielded a 99% accuracy after follow-up sequencing of any potential false negative/positive results (**Supplementary Table 1**).

**Table 1:** Hematologic Custom Gene Fusion Panel Probe Sets and Validation Samples.

Positive Samples in Validation Set	Hematologic Gene Fusion	# of Probe Sets	Pediatric/Adolescent Cancer Association
6	*BCR::ABL1*	7	B-ALL or CML
	*BIRC3::MALT*	8	Lymphoma
1	*CBFA2T3::GLIS2*	2	AML (megakaryoblastic)
1	*CBFB::MYH11*	6	AML
	*DEK::NUP214*	1	AML
1	*ETV6::RUNX1*	6	B-ALL
1	*FIP1L1::PDGFRA*	8	Chronic eosinophilic leukemia
	*FUS::ERG/FEV*	4	AML
6	*IKZF1* deletions	3	CML, Ph-like ALL
2	*KAT6A::CREBBP*	4	AML
7	*KMT2A*::xxx	70	infantile leukemia
	*MEF2D*::xxx	12	B-ALL
1	*NPM1::ALK*	1	ALCL
	*NPM1::MLF1*	1	AML
1	*NUP98::NSD1*	2	AML
	*NUP98*::xxx	8	T-ALL
1	*NUP214::ABL1*	9	B-ALL, T-ALL
3	*P2RY8::CRLF2*	1	Ph-like B-ALL
2	*PAX5*::xxx	46	B-ALL
2	*PICALM::MLLT10*	6	T-ALL
2	*PML::RARA*	3	AML (promyelocytic)
	*RBM15::MKL1*	2	AML (megakaryoblastic)
1	*RUNX1::RUNX1T1*	2	AML
	*RUNX1*::xxx	7	AML
	*SET::NUP214*	2	AML, T-ALL, B-ALL
1	*TCF3::PBX1*	2	B-ALL
	*TCF3/4*:xxx	6	B-ALL
1	xxx::*ALK*	8	ALCL, histiocytic
11	xxx::*ABL1*/*ABL2*	35	Ph-like B-ALL, T-ALL
1	xxx::*CSF1R*	5	Ph-like B-ALL
	xxx:*FGFR1*	17	B-ALL, T-ALL, AML
7	xxx::*JAK2*	36	Ph-like B-ALL
	xxx::*NOTCH1*	4	T-ALL
	xxx::*NUTM1*	6	B-ALL
2	xxx::*PDGFRB*	13	Ph-like B-ALL
	xxx:*RARA*	12	AML
	xxx::*RET*	3	CMML
1	xxx::*ZNF384*	16	B-ALL

### 2.2 Positivity cutoffs for gene fusion interpretation

During the validation, 48 clinical samples were used to determine fusion positivity cutoffs. The NanoString nCounter background counts for each probe set were averaged for all samples negative for that fusion. A sample was considered positive for a fusion probe set if the counts for the sample were more than 5 standard deviations (SD) above the negative sample average, based on the validation data to allow detection of all known positives, without false positives. Samples with counts more than 3 SD above the average were marked for further evaluation and potential for repeat analysis with more or less RNA.

### 2.3 Processing and nucleic acid extraction of leukemia samples in SSA

Bone marrow or peripheral blood from patients with suspected new or relapsed leukemia were ficoll-separated using SepMate™-15 tubes (StemCell Technologies, Vancouver CA) and Lymphoprep™ (StemCell Technologies, Vancouver, CA) onsite in the local laboratories in Uganda or Malawi. An aliquot of up to 10^7^ cells was used for extraction using the AllPrep DNA/RNA AllPrep mini kit (Qiagen, Hilden, Germany) with nucleic acids quantified on a NanoDrop™, Thermo Fisher Scientific, Waltham, MA, USA).

### 2.4 NanoString nCounter gene fusion analysis of SSA samples

NanoString nCounter Sprint instruments were installed in Uganda and Malawi by a local distributor. Technologists (OM, SM, KU) were trained on maintenance of the instrument and performance of the assays. The gene fusion analysis was performed in-country following manufacturer’s instructions. Briefly, 100 ng of RNA Heme 1 or Heme 2 probes sets (Integrated DNA Technologies, Coralville, IA, US), Elements Core and Extension TagSets (NanoString Technologies, Seattle, WA, USA), and Hybridization Buffer (NanoString Technologies, Seattle, WA, USA) were mixed and hybridized at 67°C for 20 hours. Samples were loaded onto a NanoString Sprint cartridge and processed on the NanoString Sprint instrument (run time 6 hours). Data were analyzed using the nSolver 4.0 software (approximately 5 minutes), followed by pasting of the data into the interpretation template (see below). The 12 samples can then be interpreted in 15–30 minutes. Total time from RNA extraction to result can be as little as 48 hours with batching of samples (12 per run) most cost effective.

## 3 Results

### 3.1 Gene fusion analysis of suspected leukemia samples in Malawi and Uganda

Data from each NanoString Sprint run (12 samples) was viewable in a spreadsheet containing approximately 250 rows and 15 columns. Given the challenge to review that volume of data quickly and accurately, analysis templates were created to allow data to be easily and consistently analyzed at the SSA sites. The templates were color coded to indicate fusion probe sets highly suggestive of a positive result (>5 SD above the average background for the probe set) or those that may require further review or repeat testing (>3 SD above the average background). For each sample, quality of the assay and RNA were assessed using positive and negative assays controls included in the NanoString hybridization reagents and housekeeping genes included in the probe set design. Selected sample data from a Heme 1 gene fusion run in Malawi are shown in **Table 2**.

**Table 2:** Example Data from a Heme 1 gene fusion assay in Malawi.

Probe set	5	6	8	15	16	22	24	24C	IVS-020		
POS_A - internal NS control	36825	34352	29274	36457	34254	21268	17649	35	17068		
POS_B- internal NS control	11933	10643	9357	11570	10713	6649	5447	67	5339		
POS_C- internal NS control	3254	3014	2613	3199	3095	1884	1555	29	1535		
POS_D - internal NS control	778	751	611	735	710	461	353	31	354		
POS_E - internal NS control	232	238	189	248	208	147	101	72	128		
POS_F - internal NS control	80	50	48	54	51	26	30	12	31		
NEG_A - internal NS control	11	14	5	9	4	2	7	52	2		
NEG_B- internal NS control	4	4	3	6	7	5	5	16	1		
NEG_C- internal NS control	10	5	10	6	6	4	5	76	2	POSITIVITY CUTOFFS	
NEG_D- internal NS control	6	1	3	2	6	2	1	39	2		
NEG_E- internal NS control	7	5	4	10	6	6	2	43	4	AVE + 3 SD	AVE + 5 SD
NEG_F- internal NS control	18	19	6	2	9	8	8	27	19		
BCR(ex1):ABL1(ex3)	8	9	1	5	8	2	5	27	3	21	28
BCR(ex1):JAK2(ex15)	8	11	7	4	5	3	4	30	3	15	21
BCR(ex14):ABL1(ex2)	8	6	5	15	5	5	3	64	1	18	25
CBFB(ex5):MYH11 (type A)(ex33)	12	23	9	10	8	10	4	24	7	21	28
CBFB(ex5):MYH11 (type B)(ex31)	11	11	10	4	4	5	9	48	1	20	29
EBF1(ex15):PDGFRB(ex11)	14	15	5	8	3	825	3	10	10	19	27
ETV6(ex3):RUNX1(ex3)	15	14	13	8	16	6	12	16	5	20	28
ETV6(ex4):JAK2(ex16)	13	10	8	14	5	8	5	27	7	25	35
PML(ex6):RARA(ex3)	14	9	9	5	10	5	10	82	44	22	30
RBM15(ex1):MKL1(ex4)	14	17	3	249	9	7	2	68	7	19	26
RUNX1(ex6):MECOM(ex2)	25	11	11	14	12	5	12	48	7	36	50
RUNX1(ex6):RUNX1T1(ex2)	373	20	23	46	9	16	10	45	12	94	142
TCF3(ex16):PBX1(ex3)	12	20	3	9	3	24	272	10	19	71	104
B2M Housekeeping	31726	71951	10283	30391	4801	65454	8316	34	53889		5316
EEF2 Housekeeping	13522	10173	1509	10451	637	10099	2531	36	12455		920
GUSB Housekeeping	849	1287	134	664	66	778	104	23	2358		97
PGK1 Housekeeping	3603	5680	1302	2293	620	5321	866	27	31558		683
TBP Housekeeping	143	191	48	273	F	411	67	17	863		27
Interpretation	*RUNX1:: RUN1T1*	likely negative; repeat with more RNA to check *CBFB:: MYH11*	negative sample	*RBM15:: MKL1*	failed RNA; repeat with more RNA	*EBF1:: PDGFRB*	*TCF3:: PBX1*	failed RNA: (all positive controls for assay failed)	*PML:: RARA* positive control sample		

Each column (5 through IVS-020) represents an RNA sample. The POS A-F and NEG A-F probe sets are NanoSring (NS) assay controls that are included in the Elements reagents. A partial list of the fusion probe sets is shown in bold. Brown or yellow cells are conditional formatting on the interpretation template to indicate samples above the positivity cutoffs with yellow (5 SD above the average background for the probe set) as a strong indicator of positivity and brown as a flag for additional review needed. The five probe sets for housekeeping genes are used to assess the quality of the RNA with green for adequate and red for inadequate (built in as conditional formatting in the review template). To be considered negative for fusions, a sample must be negative for the fusion probe sets and green for at least 3 of the housekeeping probe sets.

Samples for the initial SSA studies included 126 suspected leukemia samples that had adequate quantity of RNA to perform the gene fusion testing (at least 50 ng of RNA for the assay, although 100 ng was used whenever possible). Most samples were run on both Heme 1 and Heme 2 custom gene fusion panels and analyzed blindly, without knowledge of flow cytometry results. In more recent runs, a more cost-effective means of reflex testing was utilized, where the Heme 2 panel was tested only if the Heme 1 panel was negative. Samples were called negative for fusions only if RNA quality was adequate (at least 3 of 5 housekeeping genes above the cutoff). Repeat testing was performed using more RNA (if the sample was negative but failed RNA quality using 100 ng) or less RNA (if the sample had high background with positives across numerous probe sets), as needed.

### 3.2 Confirming the accuracy of gene fusion results

**Figure 1:**
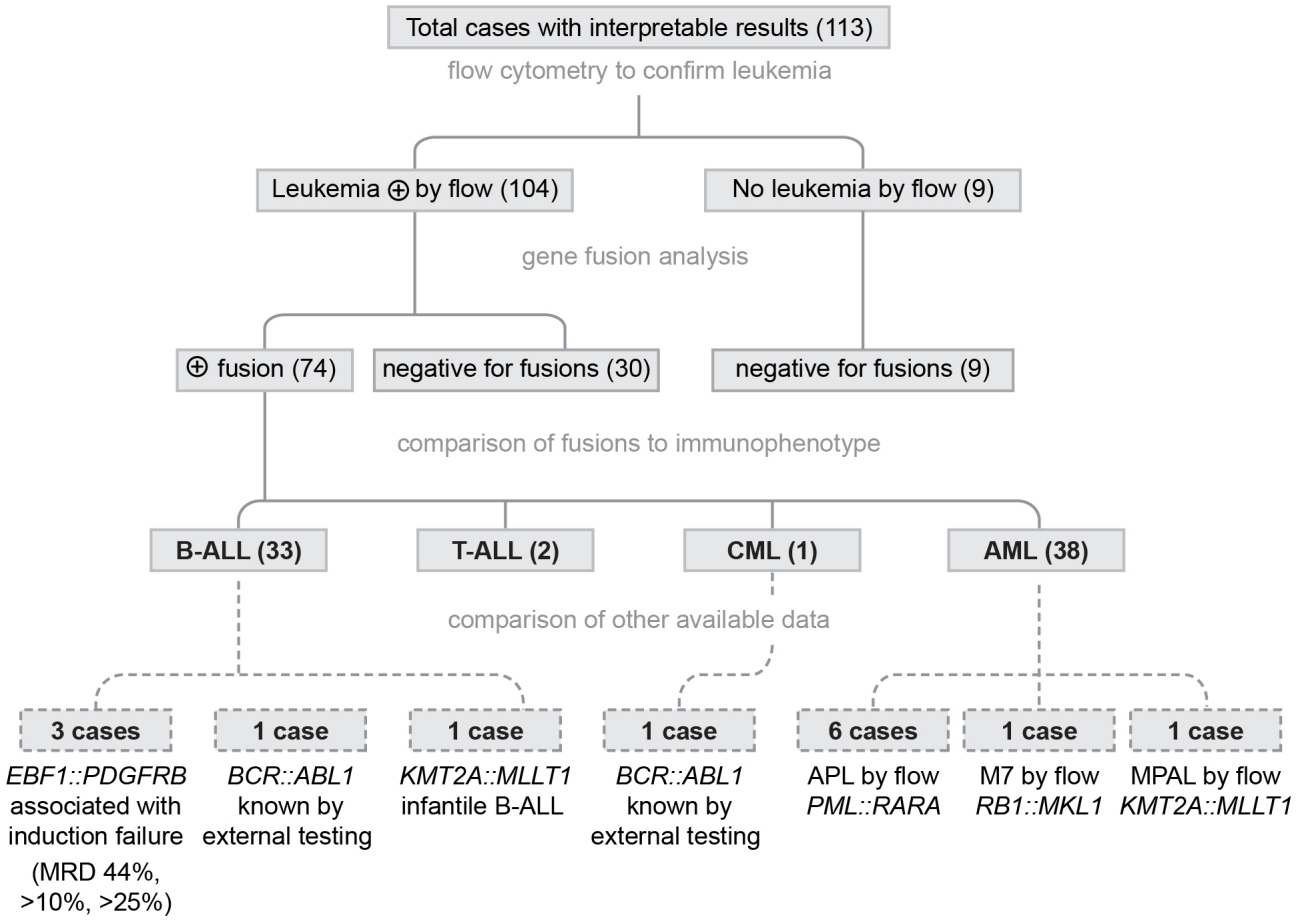
Evidence for Accuracy of the SSA Gene Fusion Results. All samples underwent molecular testing without knowledge of flow cytometry results. Nine cases were negative for leukemia by flow cytometry (all were negative for fusions). 104 leukemia cases were characterized as B-ALL (33), T-ALL (2), CML (1), or AML (38). After unblinding the flow cytometry results, all fusions results were consistent with the immunophenotype. Additional data were available for 14 cases and all data supported the accuracy of the fusion data.

**Figure 2:**
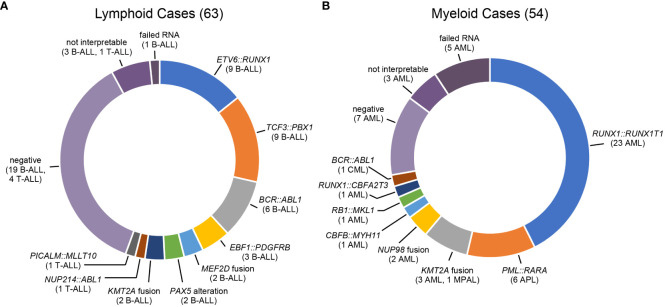
Gene Fusion Testing Results for SSA Leukemic Samples. **(A)** Lymphoid leukemias. *KMT2A* partners: *MLLT1* (1 case), *MLLT3* (1 case); *MEF2D* partners: *BCL9* (1 case), *HRNPUL1* (1 case); *PAX5* alterations: *PAX5* internal tandem duplication (1 case), *PAX5::ZCCHC7* (1 case) **(B)** Myeloid leukemias. *KMT2A* partners: *AFF1* (1 MPAL case), *AFDN* (1 case), *MLLT10* (1 case), *USP2* (1 case); *NUP98* partners: *KDM5A* (1 case), *NSD1* (1 case).

Very few leukemia cases in SSA are characterized by cytogenetic or molecular genetic testing, so genomic results were not available for comparison in most cases. To assess the accuracy of the results, the gene fusion calls were compared to flow cytometry results, including presence or absence of leukemic cells and immunophenotype (**Figure 1**). As a first step to confirming the accuracy of the fusion results, the identified fusions were compared to the fusions associated with the immunophenotype based on flow cytometry. There is some overlap in the fusions found in ALL and AML (e.g. KMTA rearrangements) and the BCR: ABL1 fusion is observed in both pediatric B-ALL and CML. However, most fusions are characteristic for a subtype of leukemia (B-ALL, T-ALL, AML, or CML). All fusion results were consistent with the flow cytometry immunophenotype. To further assess accuracy of the gene fusion testing, additional clinical or laboratory information was available for 14 cases. Three B-ALL cases were positive for the *EBF1*::*PDGFRB* fusion. This fusion is associated with Ph-like ALL and characterized by induction failure (23). The end of induction flow cytometry minimal residual disease results for these cases confirmed 44%, >10%, and >25% blasts, consistent with induction failure. One case was an infant with B-ALL that was positive for the KMT2A::MLLT1 fusion commonly found in infantile B-ALL. Two cases (1 B-ALL and 1 CML) were positive for the *BCR::ABL1* fusion by NanoString analysis. Both cases had prior external molecular analysis consistent with the *BCR::ABL1* fusion. Eight cases had a flow immunophenotype consistent with a more specific subtype. Six had a *PML::RARA* fusion, consistent with their diagnosis of acute promyelocytic leukemia (APL). One case was an infantile AML was consistent with an AML M7 megakaryoblastic leukemia immunophenotype (CD61+, CD34-, HLA-DR-) and a characteristic *RB15::MKL1* fusion was identified (24). One case was considered to be mixed-phenotype acute leukemia (MPAL) for flow cytometry and showed a *KMT2A::MLLT1* gene fusion. MPAL with *KMT2A* rearrangement is now described as a distinct entity for the World Health Organization (25). Thus, additional information available for these 14 cases further supported the accuracy of the gene fusion testing. The fusion results for 117 confirmed leukemia cases included 74 positive for a gene fusion, 26 negative cases, 7 cases with results that could not be interpreted, and 6 cases that failed RNA quality (**Figure 2** *)*. As noted previously, little has been published about the leukemia subtypes in SSA. Although case numbers in this study were relatively low, and did not represent a consecutive series of cases, the general trends of fusions identified (relatively high frequency of *RUNX1::RUNX1T1*, *PML::RARA*, and *BCR::ABL1*) were consistent with comparisons of African American and white patients in a study of AML subtypes in the United States (26).

## 4 Discussion

This initial study supports implementation of gene fusion testing in real-time for new or relapsed pediatric leukemia cases in SSA using the NanoString platform and custom gene fusion assays. This testing would impact more than 60% of newly diagnosed leukemia patients’ diagnoses, a significant percentage as a first step into molecular testing. All testing for this study was performed by trained technologists in Uganda and Malawi, and standard operating procedures have been implemented for all steps of the process. Review of fusion results will occur with knowledge of leukemic content and immunotype available from flow cytometry results of the sample. Positivity cutoffs were modified for in-country results, as both instruments in SSA had lower background counts than the instrument used for validation testing in the US. Prospective testing in SSA will allow for implementation of risk adapted and/or targeted therapy, to maximize chances of cure. Gene fusion results obtained at the SSA sites during the initial years of patient testing will be evaluated to identify the most common fusions in the SSA pediatric cancer population and better understand the genomic landscape of SSA pediatric leukemias. This analysis will also allow reduction of the hematologic gene fusion assay to a smaller number of fusions, making the assay more cost-effective (approximately $150 vs. $250 currently) without decreasing detection rate. These reagent costs remain lower than a targeted RNA fusion panel via next-generation sequencing (approximately $500 for library preparation reagents), although only known fusions can be detected with the NanoString panels. A project with collaborators in Malawi allows for low-pass RNA sequencing using the Nanopore Minion for approximately $100 in reagent cost, but the results are best for classification of leukemias by gene expression and not fusion identification. Documented fusion results are a requirement for access to some targeted drugs including imatinib at the sites in SSA. The NanoString fusion assay is only intended for diagnostic samples and not for monitoring MRD. The sites in SSA are using flow cytometry for MRD analysis, a much more sensitive test compared to NanoString (due to direct measurement of RNA fusion molecules without an amplification step in the NanoString assay).

### 4.1 Future directions

In addition to fusion analysis, the Global HOPE team is initiating studies of copy number detection, including gene amplification, on the NanoString nCounter Sprint platform using the NanoString Cancer CNV 2.0 assay with additional probes added based on relevance to pediatrics tumors. Gains and losses of whole chromosomes (a critical next step for leukemia diagnosis) can be detected using the CNV probes, but interpretation needs to be optimized to address frequent aneuploidy observed in pediatric tumors. A pediatric solid tumor custom fusion panel has also been designed and validated. Implementation in SSA will allow for more rapid and precise diagnosis. Histopathologic review of cases often requires weeks to months for a diagnosis in SSA and often does not include immunohistochemistry due to limited resources and patient lack of ability to pay. Identification of a specific gene fusion, e.g. *PAX3::FOXO1* for rhabdomyosarcoma, would lead to a precise diagnosis without the need for extensive immunohistochemistry. In addition, the copy number analysis will allow for the rapid detection of gene amplification in specific pediatric tumors (e.g. *MYCN* in neuroblastoma). Initial proof of principle experiments confirmed that it will be straightforward to detect gene amplification using copy number variation (CNV) probes.

### 4.2 NanoString technology in low resource settings

The possible uses of the NanoString nCounter as a cost-effective diagnostic method in low-resourced setting is starting to be realized due to its ability to be used for clinical and research purposes in numerous disease categories. An increasing number of studies in LMICs describe applications to adult and pediatric cancers including lung (27), breast (28), gastric (29), colon (30) medulloblastoma (31, 32), and Ph-like ALL (33).

### 4.3 Ongoing challenges for molecular testing in SSA

The biggest challenges around diagnostic testing in SSA are reagent cost and access, instrument service availability, and shipping of cold chain reagents. Most Western companies utilize local distributors in SSA to supply reagents and instrument service. While allowing for more rapid response time on comparable time zones, these arrangements often lead to reagent stock-outs and extended instrument downtime. In addition, costs are typically elevated to account for secondary distribution. Most testing in pathology is not covered by government funding and therefore requires patients to pay (usually well outside their ability) or philanthropic support. To address these issues, Global HOPE has been purchasing reagents in the US, and shipping to the sites in SSA. Shipping at multiple temperature (room temperature, 4 °C, and frozen) greatly increases the cost of the reagents and creates funding challenge and is not sustainable in the long term. Global HOPE continues to work with diagnostic companies to emphasize the needs and challenge of high quality diagnostics in low-resource settings and to work to create access programs for compassionate pricing. Such partnerships are critical to reducing the extreme disparities in patient care and outcomes.

### 4.4 Summary

Current pediatric cancer diagnostics in Sub-Saharan Africa (SSA) lack the specificity necessary to inform the application of modern curative treatment regimens, such as distinguishing between subtypes of pediatric ALL or AML. Validated custom gene fusion panels were implemented at two Global HOPE-affiliated sites in SSA. The testing required minimal technical time, was easy to perform, and results were consistent with other known data such as flow cytometry. Implementing the diagnostic use of this platform to prospectively test patients in real time will greatly impact the ability to treat pediatric cancer in SSA by providing a more precise diagnosis. Global HOPE’s partnerships with Teva and Direct Relief have allowed SSA access to an increasing number of therapeutic options (34). As the available drugs expand to include more targeted therapies, molecular genomic analysis will be critical to identify the appropriate therapy for each patient. Thus, the effect of implementing molecular diagnosis will continue to grow with time. This work will also allow establishing training in molecular and pediatric pathology in low resource settings, a critical need for long-term sustainability and reducing inequities of pediatric cancer care.

## Data availability statement

The original contributions presented in the study are included in the article/**Supplementary Material**. Further inquiries can be directed to the corresponding author.

## Ethics statement

The studies involving humans were approved by Baylor College of Medicine Institutional Review Board. The studies were conducted in accordance with the local legislation and institutional requirements. Written informed consent for participation in this study was provided by the participants’ legal guardians/next of kin.

## Author contributions

JG-F: Writing – review & editing, Writing – original draft, Visualization, Validation, Supervision, Project administration, Methodology, Investigation, Funding acquisition, Formal analysis, Data curation, Conceptualization. FL: Writing – review & editing, Validation, Methodology, Investigation. OM: Writing – review & editing, Methodology, Investigation, Data curation. SM: Writing – review & editing, Methodology, Investigation, Data curation. KU: Writing – review & editing, Methodology, Investigation, Data curation. RN: Writing – review & editing, Investigation, Funding acquisition. EH: Writing – review & editing, Validation, Methodology, Investigation. DL-T: Validation, Writing – review & editing, Resources, Investigation. KF: Writing – review & editing, Validation, Resources, Investigation. AR: Writing – review & editing, Validation, Resources, Investigation. CA: Writing – review & editing, Resources, Funding acquisition. DP: Validation, Writing – review & editing, Resources, Project administration, Funding acquisition. RM: Writing – review & editing, Project administration, Investigation, Funding acquisition, Data curation. NO: Writing – review & editing, Resources, Investigation, Funding acquisition, Data curation. PW: Writing – review & editing, Investigation, Funding acquisition, Data curation.
