# Supplementary material for: Rapid gene fusion testing using the NanoString nCounter platform to improve pediatric leukemia diagnoses in Sub-Saharan Africa
Source: Front Oncol. 2024 Jun 13;14:1426638. doi: 10.3389/fonc.2024.1426638 (PMC11208450; doi:10.3389/fonc.2024.1426638)
Supplement: Supplementary file 1 [file Table_1.docx]

Supplementary Table 1. Custom Hematologic Gene Fusion Panel Validation

| **NanoString (NS) Custom Panel** | Heme 1 | Heme 2 |
| --- | --- | --- |
| **Total Samples Tested** | 61 | 48 |
| **Unique Samples Tested** | 50 | 28 |
| **Inevaluable Samples (very high background, insufficient tumor RNA)** | 4 | 1 |
| **Evaluable, unique samples Expected Pos/Neg** | 40/6 | 15/12 |
| **Evaluable, unique samples where NS matched expected prior to confirmations** | 42/46 | 23/27 |
| **Potential False Negatives** | 1 | 1 |
| **True False Negatives by NS** | 0 | 1 |
| **Potential False Positives** | 3 | 3 |
| **True False Positives by NS** | 0 | 0 |
| **Evaluable, unique samples correct by NS after RT-PCR/Sanger confirmations** | 46/46 (100%) | 26/27 (96%) |

The custom fusion panels were validated in the Department of Pathology at Texas Children’s Hospital using residual, deidentified pediatric cancer samples with known fusion status determined by previous clinical testing, as well as commercially available RNA controls (Invivoscribe, San Diego, CA, USA and SeraCare Life Sciences, Milford, MA, USA). The fusions in the panel, number of probe sets per fusion, and the number of samples positive for the fusion included in the validation set are shown in **Table 1**. The validation design included samples to test a variety of fusions, different breakpoints for the same fusion, robustness between technicians, precision across runs, sensitivity of detection (approximately 10% blasts for high expressing fusions), and ease of performance.

Samples with results that did not match the known fusion were tested by RT-PCR and/or Sanger sequencing. For the Heme 1 panel, two cases were positive for Ph-like ALL fusion and an *IZKF1* deletion by NanoString testing. Neither case had previous testing for an *IKZF1* deletion, but both *IKZF1* deletions were confirmed by RT-PCR. One case had a previously unknown fusion confirmed by RT-PCR and one did not have the “known” fusion confirmed by RT-OCR. For the Heme 2 panel, there was one confirmed Ph-like ALL case that was a true false negative due to low expression. In the original testing of the sample at the Children’s Oncology Group reference laboratory, the fusion could only be detected by targeted NGS, but not by NanoString fusion analysis. Three cases were positive for Ph-like ALL fusion and an *IZKF1* deletion by NanoString testing. Neither case had previous testing for an *IKZF1* deletion, but both *IKZF1* deletions were confirmed by RT-PCR.
